# An analysis of 67 RNA-seq datasets from various tissues at different stages of a model insect, *Manduca sexta*

**DOI:** 10.1186/s12864-017-4147-y

**Published:** 2017-10-17

**Authors:** Xiaolong Cao, Haobo Jiang

**Affiliations:** 1 Department of Biochemistry and Molecular Biology, Oklahoma State University, Stillwater, OK 74078 USA; 2 Department of Entomology and Plant Pathology, Oklahoma State University, Stillwater, OK 74078 USA

**Keywords:** Tobacco hornworm, Transcriptome, Insect genome

## Abstract

**Background:**

*Manduca sexta* is a large lepidopteran insect widely used as a model to study biochemistry of insect physiological processes. As a part of its genome project, over 50 cDNA libraries have been analyzed to profile gene expression in different tissues and life stages. While the RNA-seq data were used to study genes related to cuticle structure, chitin metabolism and immunity, a vast amount of the information has not yet been mined for understanding the basic molecular biology of this model insect. In fact, the basic features of these data, such as composition of the RNA-seq reads and lists of library-correlated genes, are unclear. From an extended view of all insects, clear-cut tempospatial expression data are rarely seen in the largest group of animals including *Drosophila* and mosquitoes, mainly due to their small sizes.

**Results:**

We obtained the transcriptome data, analyzed the raw reads in relation to the assembled genome, and generated heatmaps for clustered genes. Library characteristics (tissues, stages), number of mapped bases, and sequencing methods affected the observed percentages of genome transcription. While up to 40% of the reads were not mapped to the genome in the initial Cufflinks gene modeling, we identified the causes for the mapping failure and reduced the number of non-mappable reads to <8%. Similarities between libraries, measured based on library-correlated genes, clearly identified differences among tissues or life stages. We calculated gene expression levels, analyzed the most abundantly expressed genes in the libraries. Furthermore, we analyzed tissue-specific gene expression and identified 18 groups of genes with distinct expression patterns.

**Conclusion:**

We performed a thorough analysis of the 67 RNA-seq datasets to characterize new genomic features of *M. sexta*. Integrated knowledge of gene functions and expression features will facilitate future functional studies in this biochemical model insect*.*

**Electronic supplementary material:**

The online version of this article doi: (10.1186/s12864-017-4147-y) contains supplementary material, which is available to authorized users.

## Background

**Fig. 1:**
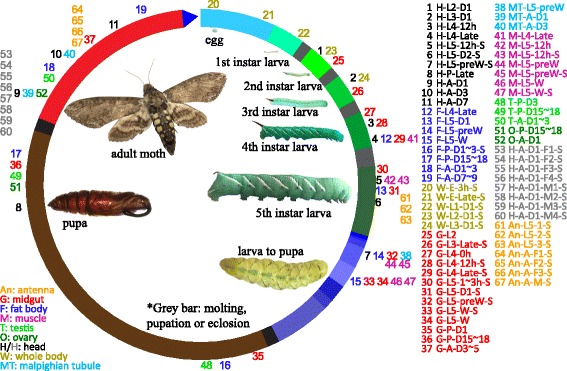
*M. sexta* life cycle and the 67 Illumina RNA-seq datasets. Bars in the circle represent different life stages of *M. sexta*, which are proportional to time periods of the insect raised with artificial diet as previously described [1]. Color-coded library identifications (1–67) are placed outside the circle at the corresponding developmental stage. The first part of the library names (on the *right*) indicates that the libraries are made from head (), fat body (), whole body (), midgut (), Malpighian tubule (), muscle (), testis (), ovary (), and antenna (). The second part indicates major stages of the insect, i.e. embryo (E), 1st to 5th instar larvae (L1 − L5), pupae (P), and adults (A). In the third part, “D” stands for day, “h” for hour, “preW” for pre-wandering, “W” for wandering, “M” for male, and “F” for female. “S” in the last part of library names indicates single-end sequencing; no “S” in the end indicates paired-end sequencing. The cDNA libraries represent the following tissues and stages: head (**H**) [**1**. 2nd (instar) L (larvae), D1 (day 1); **2**. 3rd L, D1; **3**. 4th L, 12 h (hour); **4**. 4th L, late; **5**. 5th L, D0.5; **6**. 5th L, D2; **7**. 5th L, preW (pre-wandering); **8**. P (pupae), late; **9**. A (adults), D1; **10**. A, D3; **11**. A, D7], fat body () [**12**. 4th L, late; **13**. 5th L, D1; **14**. 5th L, preW; **15**. 5th L, W (wandering); **16**. P, D1–3; **17**. P, D15–18; **18**. A, D1–3; **19**. A, D7–9], whole body (**W**) [**20**. E (embryo), 3 h; **21**. E, late; **22**. 1st L; **23**. 2nd L; **24**. 3rd L], midgut () (**25**. 2nd L; **26**. 3rd L; **27**. 4th L, 0 h; **28**. 4th L, 12 h; **29**. 4th L, late; **30**. 5th L, 1–3 h; **31**. 5th L, 24 h; **32**. 5th L, preW; **33**–**34**. 5th L, W; **35**. P, D1; **36**. P, D15–18; **37**. A, D3–5), Malpighian tubule () (**38**. 5th L, preW; **39**. A, D1; **40**. A, D3), muscle () (**41**. 4th L, late; **42**–**43**. 5th L, 12 h; **44**–**45**. 5th L, preW; **46**–**47**. 5th L, W), testis () (**48**. P, D3; **49**. P, D15–18; **50**. A, D1–3), ovary () (**51**. P, D15–18; **52**. A, D1), head () [**53**–**56**. A, D1, F (female); **57**–**60**, A, D1, M (male)], antenna () (**61**–**63**, 5th L; **64**–**66**, A, F; **67**, A, M)

As a typical holometabolous insect with five larval instars, the tobacco hornworm *Manduca sexta* has been studied for more than 70 years. Research papers on this species from 1970 to 2016 reached 3309 based on PubMed, next only to the fruit fly *Drosophila melanogaster* (47,828), the yellow fever mosquito *Aedes aegypti* (7807), the domestic silkworm *Bombyx mori* (7396), the African malaria mosquito *Anopheles gambiae* (4151), and the honey bee *Apis mellifera* (3717). As a wild insect living on solanaceous plants (e.g. tobacco and tomato) in the larval stages, *M. sexta* well represents a large group of agricultural pests in the order of Lepidoptera. It is easy to rear under laboratory conditions, grows to a weight of over 10 g on a simple artificial diet, and has a well conserved life cycle with each developmental stage in a clear time range [1]. The development includes embryo (E), five larval (L1 to L5), wandering (W), pupal (P) and adult (A) stages (Fig. 1). *M. sexta* has been extensively used as a model for research on cuticle formation [2, 3], hormonal regulation [4], neurobiology [5], lipid metabolism [6], immunity [7], and many others.

Next-generation sequencing (NGS) is a powerful technology for transcriptome analysis [8]. Up to January 1 of 2017, over 82,340 sequencing tests from about 1700 insect species were deposited in NCBI Sequence Read Archive. *D. melanogaster* and *A. gambiae* account for 41 and 17% of the sequencing runs, and 28 and 24% of the sequenced bases, respectively. Most of the sequencing experiments fulfilled their specific research goals using whole insects or major body parts. Others analyzed specific parts of the insects, such as different regions of the digestive tract [9]. In comparison, the nucleic acids of other insects have been much less sequenced. One of the exceptions is *M. sexta*, whose cDNA libraries of fat body, hemocytes, and other tissues were sequenced in 2005 when NGS became commercially available [10]. Over the years, nucleic acids from this insect have been sequenced many times to understand digestion [11], immunity [12, 13], sex-biased gene expression [14], chemosensory perception [15], and microRNA regulation [16–18].

Recently, a draft genome sequence of *M. sexta* was published along with extensive RNA-seq data from 52 combinations of tissues and stages [19], providing an overview of the genome in action and support for evidence-based gene modeling. The expression data were also analyzed as a part of the companion papers focusing on cuticle proteins [2], chitin metabolism enzymes [3], chitin-binding proteins [20], pathogen recognition proteins [21, 22], serine proteases [23], immune signal transducers [24], and immune effectors [25]. Nevertheless, the RNA-seq data were neither thoroughly described nor systematically analyzed to provide an overview of gene expression in tissues and life stages. We published a paper describing the development of methods to incorporate the extensive RNA-seq data into genome annotation, evaluate gene models in the MAKER2, Cufflinks, Oases and Trinity assemblies, and select the best ones to constitute MCOT1.0 [26]. After manual annotation through global cooperation of researchers, the Official Gene Set 2.0 (OGS2.0) were released in 2014 with the genome paper published in 2017 [19]. The MCOT1.0 models (ftp://ftp.bioinformatics.ksu.edu/pub/Manduca/) contain protein-coding genes uncovered by OGS2.0 [26] and, since both gene sets are biased toward protein-coding genes, a large portion of the noncoding genes were not analyzed. Additional Illumina RNA-seq reads from head and antenna (15 datasets) are publicly available for fuller use in the discovery of tissue-specific genes beyond the goals of the original studies [14, 27]. Due to these reasons, we performed an overall transcriptome study of the 67 datasets and report our findings here.

## Methods

### Data and program acquisition

The final version of *M. sexta* genome assembly (Msex1.0) and gene models in *M. sexta* official gene set 2.0 (OGS2.0) were downloaded from the *M. sexta* workspace at National Agricultural Library (https://i5k.nal.usda.gov/Manduca_sexta) [28]. The MCOT1.0 gene models were generated from our previous work [26]. The RNA-seq datasets were downloaded from NCBI Sequence Read Archive with accession numbers listed in (Additional file 1: Table S1) or previously acquired from Dr. Gary Blissard at Cornell University. Trimmomatic (0.32) [29], SAMtools (1.3.1) [30], Bowtie2 (2.2.6) [31], TopHat (2.0.12) [32], Cufflinks (2.2.1) [33], STAR (2.5.2a) [34], TransDecoder (3.0.0) (https://github.com/TransDecoder), RSEM (1.2.29) [35], BLAST+ (2.2.30) [36], and tRNAscan-SE (1.3.1) [37] were downloaded from their official sites and installed on a local supercomputer. MeV (Multiple Experiment Viewer 4.9.0, http://mev.tm4.org/) and Cluster 3.0 (by Michael B. Eisen) were installed in a local computer.

#### Reads alignment and generation of Cufflinks4.0 gene models

As described previously [26], Cufflinks4.0 was generated using Cufflinks (2.2.1) [33] and reads from the 67 RNA-seq libraries. The reads were first trimmed with Trimmomatic to remove adaptors and low quality bases with the setting “SLIDINGWINDOW:4:20 LEADING:10 TRAILING:10 MINLEN:50”. Trimmed single and paired reads in each library were aligned to the genome by TopHat. Cufflinks and Cuffmerge were used as described before [26] to generate and combine GTF files to make final gene models in Cufflinks4.0. The Cufflinks4.0 GTF file was used to build the genome and assist STAR alignment. Trimmed reads were also aligned to the genome by STAR in the 2-pass mapping mode to ensure maximum alignment. Unmapped reads were separately stored for further analysis.

### Classification of reads as mitochondria, rRNA, protein-coding, and noncoding gene fragments

Gene models in Cufflinks4.0 were analyzed and classified into four groups: mitochondrial, rRNA, mRNA, and noncoding. Genes in the three scaffolds AIXA01038378.1–80.1 were mitochondrial. Since the four scaffolds AIXA01021581.1–2.1, 01032915.1 and 01037114.1 were mostly rRNA fragments, reads matching these were classified as rRNA. Genes were considered as protein-coding, if any of their transcripts can be translated to >100-residue proteins using TransDecoder. Genes did not meet the criteria were classified as noncoding. The read count and fragments per kilobase per million mapped reads (FPKM) value of each gene in each library were calculated using RSEM (1.2.29) [35]. Read counts of the four gene categories were summed for figure plotting.

### Coverage of the genome mapped with reads and calculation of gene expression

The number of reads mapped to each scaffold of the genome was extracted using idxstats function of SAMtools [30]. The sequence depth for each base of the genome in each library was extracted using SAMtools’ depth function. Transcribed genome regions were obtained by counting the non-zero numbers in each library. Transcripts in Cufflinks4.0 were translated with TransDecoder in the genome-guided mode, which yield a GTF output file with coding sequence (CDS), mRNA, gene, and untranslated region locations. The length of a gene transcript was defined as the maximum distance between its exons’ edges. Parts of the genome are transcribed, and a part of a transcript is a protein coding region (i.e. CDS). For calculation of transcript or CDS ratio in the genome, a base of the genome was considered transcribed if it was within any exon or CDS region of Cufflinks4.0, regardless of its being on the positive or negative strand of the exon. The GTF files of *D. melanogaster* and human were downloaded from Flybase (ftp://ftp.flybase.net/releases/FB2017_03/dmel_r6.16/gtf/dmel-all-r6.16.gtf.gz) and NCBI (ftp://ftp.ncbi.nlm.nih.gov/refseq/H_sapiens/annotation/GRCh38_latest/refseq_identifiers/GRCh38_latest_genomic.gff.gz), respectively. Gene, exon and CDS were defined in their GTF files. For scaffolds longer than 200 kilobases (the 3 mitochondrial and 4 rRNA scaffolds were less than 11 kilobases), mapping depth for each base was normalized and shown as a BPKM (bases per kilobase per million mapped bases) value [38], which is equal to the number of bases mapped to one base out of one billion mapped bases. All the bases in the genome were sorted based on BPKM values and divided into 19 groups for each library, which are top 1–400, 401–800, 801–1600, 1601–3200, … 400 × 2^n^ + 1 to 400 × 2^n + 1^, where n equals 0 to 17 for groups 2 to 19, respectively. BPKM values were 0 for bases below 400 × 2^18^. Average BPKM value and z-score for each group were calculated across the 67 libraries; Percentages of bases in each group were calculated for plotting Fig. 3d. To calculate gene expression, trimmed reads were aligned to OGS2.0, MCOT1.0 and Cufflinks4.0 in different runs using RSEM [35]. The FPKM values and expected read counts for each gene or transcript in each library were summarized in an intermediate table (data not shown) prior to further analysis.

### Unmapped reads analysis

After STAR alignment to Msex1.0, the unmapped reads in each library were used as queries to search the NCBI non-redundant nucleotide database (2016–07-18) downloaded to the local supercomputer. For the BLASTN search, e-value threshold was set to 10^−6^ and the top hit was kept in the hit table. Reads with hits were classified into 7 categories based on their subject sequences and then counted in each category for ratio calculation. The categories are: 1) rRNA (“ribosomal RNA” or “rRNA”), 2) mitochondrion (“mitochondri”), 3) phage (“phage”), 4) *M. sexta* (“M. sexta”, “manduca” or “sexta”), 5) *E. coli* (“Escherichia coli”, “e.coli” or “*E. coli*”), 6) *Oryza* (“oryza”), and 7) others, where keywords in parentheses were used for classification and counting in each library with simple python scripts. The search terms, listed along with the group names and subject sequences, were grouped by matching these keywords in a case-insensitive manner in the order of the group names, meaning a sequence of *M. sexta* rRNA would be grouped to rRNA instead of *M. sexta*.

### OGS2.0 gene naming

Proteins from OGS2.0 were used as query sequences in a BLASTP search against NR database of NCBI in a local supercomputer with “hit-table” as the output format. Subject sequences which had at least one hit with identity >25%, alignment length > 50, e-value <10^−6^ and bit score > 100 were considered homolog of the query sequences. Query genes were named by the best matched homologous sequences by retrieving gene information with the accession number of subject sequences from NCBI with Python scripts. The file linking the accession number with gene ID (gene2accession.gz) and the file with details information for genes (gene_info.gz) can be downloaded from NCBI (ftp://ftp.ncbi.nlm.nih.gov/gene/DATA/). Highly expressed genes were manually examined to ensure accuracy.

### Library-correlated genes and interlibrary comparisons

The same definition and method used by Li et al. [39] were used to identify library-correlated genes and to compare different libraries. Basically, z-scores [z_i_ = (x_i_-μ)/s] were calculated from the FPKM values of each gene in all the libraries, where x_i_ is the FPKM value in a specific library, μ and s are the average FPKM and its standard deviation. Genes with z-score > 1.5 and FPKM value >1 were considered to be correlated with that library. Pairwise library comparisons were done by testing dependence of the correlated genes. Libraries X and Y are two samples of a population; null hypothesis is that they are independent. Suppose total gene number is n, correlated genes in X and Y are x and y, and they share some library-correlated genes. If X and Y are independent, c (for common correlated genes) equals (x × y)/(n × n), and probability for observing a higher c will decrease as the value of c increases. The probability over c values were calculated as:

$$ P={\sum}_{i=c}^{\min \left(x,y\right)}\frac{\left(\begin{array}{c}\hfill n\hfill \\ {}\hfill i\hfill \end{array}\right)\left(\begin{array}{c}\hfill n-i\hfill \\ {}\hfill x-i\hfill \end{array}\right)\left(\begin{array}{c}\hfill n-x\hfill \\ {}\hfill y-i\hfill \end{array}\right)}{\left(\begin{array}{c}\hfill n\hfill \\ {}\hfill x\hfill \end{array}\right)\left(\begin{array}{c}\hfill n\hfill \\ {}\hfill y\hfill \end{array}\right)}={\sum}_{i=c}^{\min \left(x,y\right)}\frac{x!y!\left(n-x\right)!\left(n-y\right)!}{n!i!\left(x-i\right)!\left(y-i\right)!\left(n+i-x-y\right)!}. $$

Bonferroni corrected *p*-value = *p*-value × number of pairwise comparison.

Mapping score = −log_10_(Bonferroni corrected *p*-value).

Here, the pairwise comparisons are 67 × 67 = 4489. For a mapping score > 10, the corrected *p*-value is so small that the null hypothesis can be rejected, i.e. the two libraries are considered to be dependent. The log_2_(mapping score) values were calculated for plotting Fig. 5.

### Library-correlated gene expression and gene ontology (GO) enrichment analysis

FPKM values based on OGS2.0, MCOT1.0, and Cufflinks4.0 were calculated using RSEM. Z-score were calculated based on the FPKM values. MCOT1.0 genes with bad or no match with OGS2.0 were considered as MCOT-specific genes, and noncoding genes in Cufflinks4.0 were defined as those that cannot be translated by TransDecoder to proteins longer than 100 residues. OGS2.0, MCOT-specific, and noncoding genes were combined; Genes with at least one FPKM value of >100 in the 67 libraries were selected for hierarchical clustering of the z-scores with MeV (4.9.0). The clustered genes were separated into their original three groups for plotting in Fig. 7 and Additional file 2: Figure S1. GO annotations for OGS2.0 genes were acquired by running InterProScan with BLAST2GO program [40]. Genes in Fig. 7 were divided into 18 groups based on the expression pattern. GO enrichment analysis for each group was performed with GOATOOLS [41], using all genes in Fig. 7 as background reference. The corrected *p*-values of different GO terms were calculated with the Benjamini/Hochberg method with a false discovery rate (FDR) of 0.05. Significantly enriched GO terms (corrected *p*-value <0.05) were labeled in Fig. 7.

### tRNA gene modeling and codon usage

tRNAscan-SE was used to scan the genome to identify tRNA genes under the default setting for eukaryotes. Genome-based codon usage was calculated by directly adding all codons used by protein-coding regions in OGS2.0 sequences. Transcriptome-based codon usage was calculated by first obtaining numbers of codons in the longest open reading frames of different transcripts, multiplying these numbers by FPKM values of the individual transcripts, summing them according to the 64 codons, calculating percentages of usage in each library, and averaging the percentage data across the 67 libraries.

## Results

### Overview of the 67 cDNA libraries

**Fig. 2:**
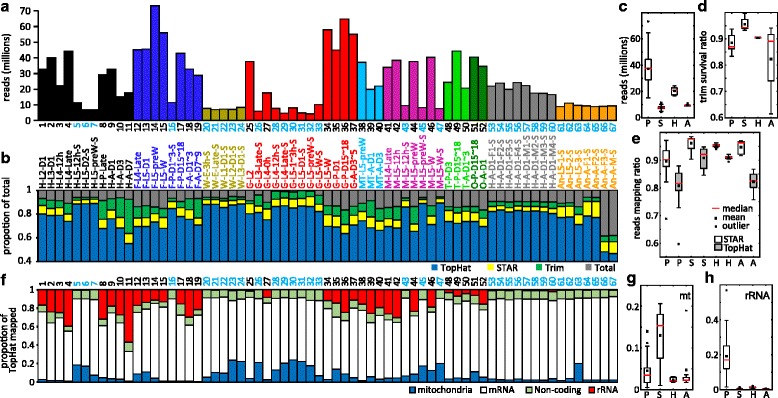
Overview of the 67 cDNA libraries. **a** Total read numbers in the libraries. As defined in Fig. 1, bar colors represent the tissue sources of libraries 1–67. Black and cyan IDs indicate the libraries were determined by paired- and single-end sequencing, respectively. **b** Up-boundaries represent percentages of the total reads after trimming (green) and mapping by STAR (yellow) and TopHat (blue), with the total reads (grey) in each library set at 100%. The library names and their color codes are the same as in Fig. 1. **c** and **d**. Box-plots of survived read numbers and percentages after trimming in categories P (paired-end, 33 of the first 52 libraries), S (single-end, 19 of libraries 1–52), H (head, single-end, 53–60), and A (antenna, single-end, 61–67). **e** Percentages of trimming-survived reads mapped to the genome using STAR and TopHat in the four categories. **f** Percentages of TopHat-mapped reads corresponding to mitochondrial (blue), protein-coding (white), noncoding (green), and rRNA (red) genes. **g** and **h** Box-plots of percentages of trimming-survived reads mapped to mitochondrial and rRNA genes in categories P, S, H and A. The first 52 libraries were sequenced as a part of the genome project [28], the next 8 were for detecting sex-biased genes expression in brain [14], and the last 7 were used to study chemosensory receptor gene expression [27]

NCBI Sequence Read Archive contains 67 *M. sexta* RNA-seq datasets (Fig. 1), representing different tissues and life stages of this insect: P for 33 **p**aired-end (read length: 100 bp) and S for 19 **s**ingle-end (51 bp)] samples sequenced as a part of the genome project [28], H for 8 **h**ead samples (single-end, 51 bp) to study sex-biased gene expression [14], and A for 7 **a**ntenna samples (single-end, 94 bp) to examine chemosensory receptor genes [27]. Names and descriptions of the libraries are shown in Fig. 1 and (Additional file 1: Table S1). The four groups of P (33), S (19), H (8), and A (7) libraries are analyzed and compared. While there is no biological replicate for libraries 1–52, four samples (i.e. G-L5-W, M-L5–12 h, M-L5-preW, M-L5-W) were analyzed by both single- and paired-end sequencing (Fig. 1). The numbers of total reads in these libraries vary greatly (Fig. 2a), ranging from 4.2 million in G-L5-preW-S (library 32) to 73 million in F-L5-preW (library 14). In general, there are many more reads in group P than in group S (Fig. 2c), with an average of 37 million versus 7.7. The variation of read numbers is the highest in group P and smallest in groups H and A, as these 15 libraries include biological replicates from the same sample types.

We generated more accurate Cufflinks4.0 gene models based on a newer version of the genome assembly Msex1.0 (with new IDs for scaffolds and three mitochondrial sequences) and quality-controlled reads from the 67 RNA-seq libraries. In the first step, reads with low quality bases were removed by Trimmomatic and reads longer than 50 were kept for further studies (e.g. unmapped read analysis) to reduce the chance of non-specific matching. Higher percentages of read survival were detected in group S (average: 96%) than in groups H (90%), P (88%), and A (82%) (Fig. 2b and d). Rates for libraries 66 and 67 (group A) were as low as 61%. We then mapped the remaining reads to the genome using TopHat and obtained Cufflinks4.0 gene models (Additional file 3 for coding transcripts; Additional file 4 for noncoding transcripts) [33]. Interested in unmapped reads, we also used STAR [34] to map the reads to the genome. Assisted by the GTF file generated from the Cufflinks program and running in the 2-pass mapping mode, STAR mapped nearly 10% more reads to the genome than TopHat did (Fig. 2e).

As anticipated, mRNA reads account for the largest portion of total reads in each library (Fig. 2f). The rRNA and mitochondrial gene reads are substantial in libraries 1–52, especially when their low gene counts are considered. This indicates that poly-A-minus RNA was not completely removed in these libraries (e.g. H-A-D7, G-L5–1~3 h–S). Higher percentages of rRNA reads were often accompanied by lower percentages of mitochondrial gene reads (Fig. 2f−h). In comparison, libraries 53–67 were mostly mRNA and noncoding RNA reads. The 33,378 genes in Cufflinks4.0 include 3 mitochondrial, 4 rRNA, 14,532 mRNA and 18,839 noncoding genes. Even though noncoding gene number is higher than coding genes in Cufflinks4.0, noncoding genes were generally shorter than coding genes [26] and their contribution to the total reads is only about 10% of that by coding genes in each library (Fig. 2f).

### Genome transcription

**Fig. 3:**
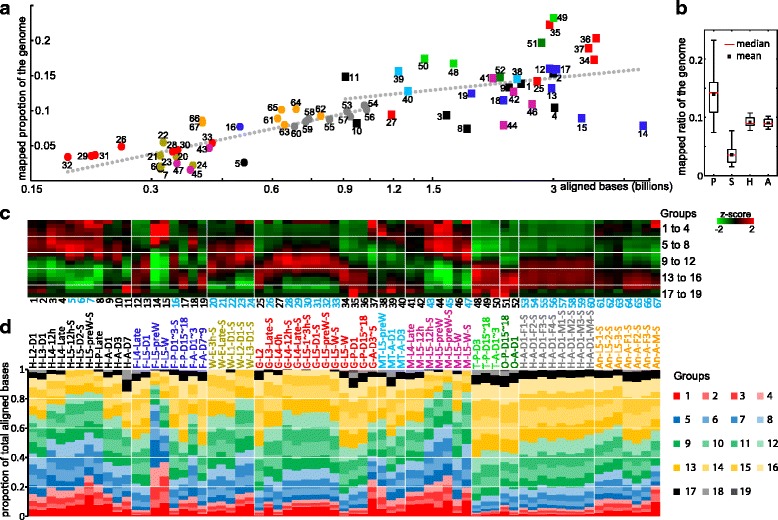
Features of gene transcription revealed by alignment of reads in the cDNA libraries. **a** Relationship between aligned bases (x-axis) and percentages (y-axis) of the genome overlaid with reads using TopHat. Each colored symbol represents one library, with their library IDs labeled (Fig. 1). Squares for paired-end libraries; circles for single-end ones. The dashed lines are linear regression of the data from the paired- and single-end libraries. **b** Box-plot of percentages of the mapped genome in library categories P (paired-end, 33), S (single-end, 19), H (head, single-end, 8) and A (antenna, single-end, 7) (Fig. 2). **c** Heatmap of z-scores in each group of base range. BPKM values were used for sorting into 19 groups. Group 1 has the highest BPKM values 1–400; Groups 2 to 19 correspond to BPKMs ranked 401–800, 801–1600, 1601–3200, … 400 × 2^n^ + 1 to 400 × 2^n + 1^, where n equals 0 to 17. The heatmap is colored based on the z-score of average BPKM in each group. Libraries with black and cyan IDs were determined by paired- and single-end sequencing, respectively. **d** Percentage of aligned bases for each BPKM group in the total aligned bases for a specific library. The library names and their color codes are described in Fig. 1

Genes in disparate parts of the genome are differentially transcribed in various tissues or life stages and their RNA products can be detected by RNA-seq technology. Based on the Cufflinks4.0 models, 51.7% of the *M. sexta* genome contains genes, 17.1% is transcribed to exons of genes, and 5.3% is protein-coding regions. The percentages are 41.6%, 9.2% and 5.1% based on OGS2.0 models for *M. sexta*, 65.4%, 24.9% and 15.9% for *D. melanogaster*, and 52.1%, 4.1% and 1.2% for human. The percentage of CDS for *D. melanogaster* is much higher than *M. sexta*, but the total DNA length is 22.8 million, similar to 22.3 million of *M. sexta* (Cufflinks4.0). Interestingly, the transcribed portion goes up to 63.9% based on the mapped reads which is much higher than 17.1% by Cufflinks4.0, and the ratios differ strikingly in different libraries, ranging from 1.5% in library 45 (M-L5-preW-S) to 23.2% in library 49 (T-P-D15~18) (Additional file 1: Table S1). While temporospatial expression certainly affects the mapped portion of genome, more RNA-seq bases generally lead to higher genome coverage (Fig. 3a). The linear regression analyses were performed for both single (S) and paired (P) end libraries to identify significant outliers. Libraries below the lines have relatively lower ratios or more highly transcribed bases; libraries above the lines have higher than usual genome coverage or are less biased toward using various parts of the genome. The testis libraries (e.g. library 49) are much higher above the line, consistent with the observation that they have fewer genes expressed at high levels [28]. In general, the mapped portion in library group P is higher than in group S, partly due to the higher number of aligned bases (Fig. 3b).

Portions of mapped genome vary dramatically across the libraries. In addition to total numbers of aligned bases, their distribution across the genome may also contribute to the variations. To test this hypothesis, we first obtained sequencing depths for each base of the genome in all libraries. As rRNA and mitochondrial reads were overrepresented in some libraries (see above), we removed the scaffolds shorter than 200 kilobases before the analysis. We then normalized the sequence depth by using the BPKM values, sorted the bases according to their sequencing depth, divided them into 19 BPKM groups from high to low, and calculated average BPKM values and z-scores for each group for comparison across the 67 libraries (Fig. 3c). Figure 3d shows the ratio of aligned bases in each group. As expected, in libraries below the linear regression line in Fig. 3a generally, more RNA-seq bases were aligned to top transcribed base groups (Fig. 3d), and the average BPKM values for top groups were higher than other libraries (more red in top base groups in Fig. 3c). For instance, top base groups of libraries 14 and 15 have higher BPKM values than most other libraries (more red than for groups 1 to 6 in Fig. 3c). In fact, 12,800 top transcribed bases (0.004% of the studied scaffolds) in groups 1 to 6 contribute 64.8% and 49.9% of aligned RNA-seq bases, respectively (Fig. 3d). For libraries 5 and 16, which have similar number of aligned bases (0.5 billion) but very different percentages of mapped genome (2.5% and 7.7%), average BPKM value of library 5 is higher than library 16 (BPKM: 3.7 × 10^5^ vs. 1.3 × 10^5^) in highly transcribed groups 1–5 (ratio: 23.6% vs. 8.5% of the mapped bases) but lower (BPKM: 3.8 vs. 6.4) in lowly transcribed groups 10–19 (ratio: 39.9% vs. 68.5%). In the midgut libraries 25–37, a clear change of high z score in BPKM ranking is observed from larva to adult (Fig. 3c). In testis and ovary libraries 48–52, base groups 1 to 10 have lower than average BPKM values (green color in Fig. 3c), while groups 13 to 19 have higher BPKM values (red color in Fig. 3c), which is consistent with their higher positions in Fig. 3a. There are large variations in the ratio of each highly-transcribed groups: Groups 1 to 4 are top 3200 transcribed bases, which may come from a few genes, as the average length of transcripts in OGS2.0 is about 2000 bp. In other words, such genes may contribute to 20–40% of the total mRNA bases. Groups 1–12 cover top 819,200 transcribed bases, which may represent 400 genes or 2.6% of the total OGS2.0 set and, on average, they account for >63% of the aligned bases. Groups 13–16 have contributed another 32% or about 6000 genes. These results confirm the large variation in expression levels, with a few highly expressed genes contributing to a major part of the sequenced RNAs [28]. Additionally, the observed variations among the libraries suggest that lists of highly expressed genes are quite different and worth exploring.

### Unmapped reads

**Fig. 4:**
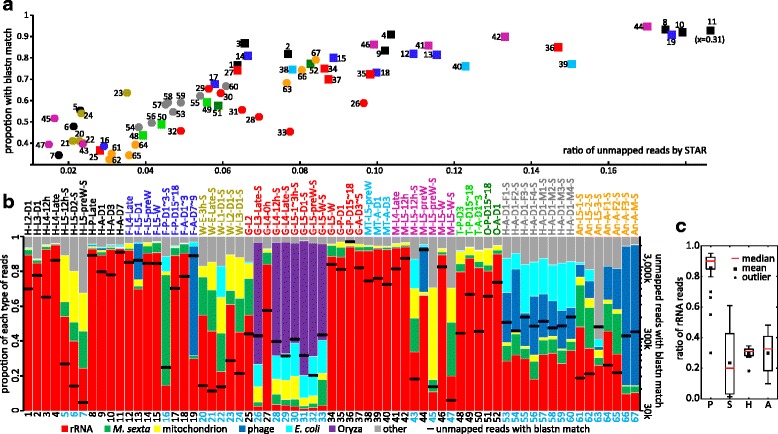
Features of the unmapped reads with BLASTN hits in the 67 libraries. **a** Relationship between ratios of STAR-unmapped reads (x-axis) and percentages of the total unmapped reads with BLASTN match (y-axis) for all the RNA-seq libraries. Each colored symbol represents one library, with their library IDs labeled (Fig. 1). Squares for paired-end libraries; circles for single-end ones. **b** Distribution (*left* y-axis) of unmapped reads with hits in the 7 categories in different colors. Black line shows the total number of unmapped reads (*right* y-axis) in a library. **c** Box-plot of percentages of rRNA reads in total unmapped reads with BLASTN hits in library categories P, S, H and A (Fig. 2). The library IDs, names, and color codes are same as in Fig. 1

During the initial gene modeling by the standard protocol of Cufflinks [33], we noticed that percentages of mapped reads were only around 60% (data not shown). Curious about ratio and composition of the unmapped reads, we controlled the read quality using Trimmomatic, mapped the reads using TopHat and then STAR in the 2-pass mode [34] with the splicing site information from Cufflinks, and improved the mapping ratio to 92.6% on average. For unknown reasons, library 11 still had a low ratio of 69% (Fig. 4a). Since unmapped reads may come from un-sequenced regions of the genome, symbionts, viruses or other sources, we searched NCBI nr/nt database (*M. sexta* OGS2.0 sequences not yet included) with the STAR-unmapped reads using BLASTN. Sequences with more than 10 reads matched were summarized in (Additional file 5: Table S2). There was a positive correlation between ratios of STAR-unmapped and BLASTN matching. Paired-end libraries had higher ratios in both than single-end ones, with the exceptions of libraries 48–51. Reads without BLASTN match, accounting for 22% of the total unmapped reads, might be low complexity ones (e.g. AT-rich sequences) or from not sequenced parts of the genome.

We divided the BLASTN hits to seven groups after initial analysis (Fig. 4b). rRNA reads accounted for 80.2% of the total (Additional file 2: Table S3), indicating their genes were incomplete in the OGS2.0. Correlating with the higher mapped ratios (Fig. 2g), library group P had higher ratio of unmapped rRNA reads (Fig. 4c), except for library 19. Libraries 19, 66 and 67 contained phage φ174 reads accounting for 62.6, 79.6, and 80.5% of the total unmapped reads – the phage DNA was used as internal positive control in DNA sequencing. While percentages for the phage reads varied a lot in the libraries, the average was 6.9% (Table S3). The mitochondrial reads (1.8% of the total) were mapped to a more complete version of *M. sexta* mitochondrion in NCBI. The *Oryza* reads (1.1%), linked with the midgut libraries (26, 28–33) of feeding larvae, probably represented plant RNA in the artificial diet; *E. coli* reads (1.7%), correlated with the midgut and certain head libraries (26, 28–33, 53–60), may represent microbiota of the midgut and foregut, as part of the head. The other head libraries (1–11) apparently were less contaminated by foregut tissues. Likely due to allelic variations, 2.8% of the total were mapped to the *M. sexta* sequences previously deposited at NCBI, including highly expressed lysozyme, apolipophorin and other genes. Other reads (5.5%) matched sequences of lepidopteran insects and other bacteria. No viral sequence was detected in this lab strain which had been established for a long period of time [28]. Insects caught in the field are often infected by viruses that are detectable by RNA-seq analyses [42].

### Comparisons of different libraries and their gene expression

**Fig. 5:**
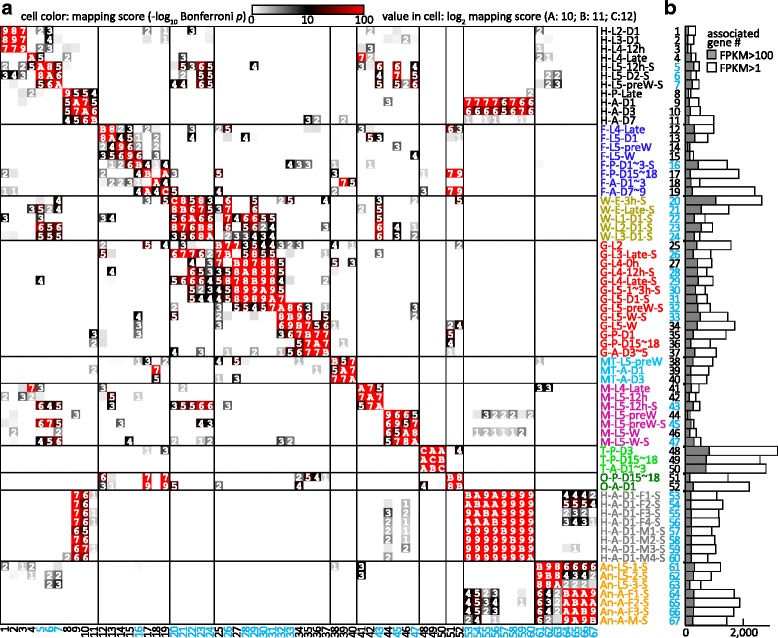
Pairwise comparison of the 67 cDNA libraries and number of library-correlated genes. **a** Mapping scores of library pairs. Value in a cell represents log_2_(mapping score). If higher than 4 (i.e. mapping score > 16), two libraries were closely similar or related. **b** Number of the correlated genes in each library, with grey bars indicating those with FPKM value >100

FPKM values and gene names of OGS2.0 are enlisted in (Additional file 6: Table S4). So are FPKM values of Cufflinks4.0 genes in (Additional file 7: Table S5) and MCOT1.0 genes in (Additional file 8: Table S6). Based on the definition of library-correlated genes (FPKM >1, z-score > 1.5) [39], 15,289 of the 15,543 OGS2.0 genes are correlated with at least one library, and the rest are expressed at low levels (FPKM <1). The correlated genes range from 200 to 3000 in each library. Early embryo, pupa, and adult generally had more correlated genes, but lower than testis (libraries 48–50) (Fig. 5b). Percentages of the highly expressed ones (FPKM >100) vary to some extent.

We compared the 67 libraries based on mapping scores using the same strategy described by Li et al. [39]. Digitized scores in the color gradient clearly showed the interlibrary relationships (Fig. 5a). As anticipated, mapping scores close to the diagonal line were much higher, indicating that the libraries with closer developmental stages from the same tissue type are more similar to each other. Some square-shaped regions of different sizes along the diagonal line overlap each other (e.g. midgut libraries 27–32, 32–35 and 34–37); others do not (e.g. head libraries 1–3 and 4–5). Gene expression (e.g. digestive enzymes) in midgut of the feeding larvae, wandering larvae, and pupal − adult stage changed progressively, with each library more similar to its neighboring libraries or developmental stages [23, 28]. Libraries from the same tissue/organ/body part but distant in life stage share fewer correlated genes and behave independently in the heatmap. Conversely, those with close developmental stages share more correlated genes (e.g. libraries 53–60 vs. 9–10, head, young adults). For antenna libraries 61–67, larvae and adults are somewhat similar, female adults (64–66) resemble female head (53–56) and, surprisingly, the male antenna library 67 is similar to female head (53–56). The whole body libraries 20–24 show higher similarity with libraries of midgut from feeding larvae, larval head and muscle. The ovary libraries 51–52 (O-P-D15~18 and O-A-D1) are highly similar to fat body libraries 17 (F-P-D15~18) and 19 (F-A-D7~9). Surprisingly, the fat body library 18 (F-A-D1~3) is most similar to the Malpighian tubule libraries 39 (MT-A-D1) and 40 (MT-A-D3). Sequencing methods seem to affect library similarity (i.e. mapping scores) for unclear reasons. Single-end muscle libraries 43, 45 and 47, but not the corresponding paired-end ones (42, 44 and 46), are highly similar to single-end head libraries 5–7.

### Highly expressed genes in the libraries

**Fig. 6:**
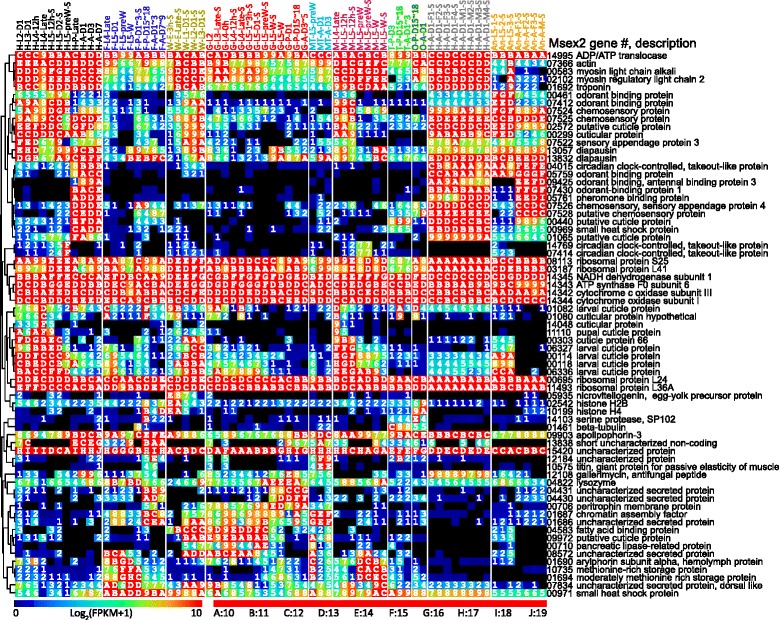
Expression profiles of 69 highly expressed genes in the 67 cDNA libraries. A non-redundant collection of the three genes with highest expression in each library are on the *right*. Their expression patterns are organized according to the results of cluster analysis (*left*). Their log_2_(FPKM + 1) values, representing mRNA levels, are shown in a rainbow color gradient in the heatmap. Library names (*top*), IDs (*bottom*), and color codes are described in Fig. 1

Genes with high FPKM values contribute a major portion of the total reads in all libraries. For the current study, we selected top 3 expressed genes in each library, removed the redundant ones, and examined expression patterns of the remaining 69 (Fig. 6). Among them, housekeeping genes (e.g. ribosomal proteins) are highly expressed in nearly all libraries. Expression of odorant-binding/chemosensory proteins are high in head and antenna. Some cuticle proteins are abundantly made by epithelial cells in the head and whole body samples.

Very high read numbers of serine protease-102 in libraries 17 and 51 (F-P-D15~18 and O-P-D15~18) suggest its role in tissue remodeling during metamorphosis in the late pupal stage (Fig. 6). Other tissue-specific proteins include titin in young adults (MT-A-D1 and MT-A-D3), histones H2B and H4 in embryo (W-E-3 h-S and W-E-Late-S), circadian clock-controlled gene products in head (H-L5-preW-S for OGS2.0 genes Msex2.07414 and Msex2.14769); adult head libraries 9–11 and 53–60 for gene Msex2.04015), lysozyme in fat body and midgut, and diapausin-4 and -13 (Msex2.13832 and Msex2.13057) in head, fat body, and other tissues. Insect development is controlled by diverse clocks, including the circadian clock [43]. The fact that circadian clock-controlled genes (Msex2.07414 and Msex2.14769) are expressed in very high levels in head of pre-wandering larvae suggests vital roles for their proteins in this and the wandering stage. The founding member of the diapausin protein family in the leaf beetle was named diapausin on the basis of stage-specific synthesis [44]. A group of 14 diapausins was later identified in the *M. sexta* genome, with diapausin-1 shown to be an antifungal peptide [25, 45]. While the high mRNA levels for diapausin-4 and -13 in fat body may contribute to the antifungal activity of hemolymph, it is interesting to note that their expression levels were even higher in head.

### Library-correlated expression of genes

**Fig. 7:**
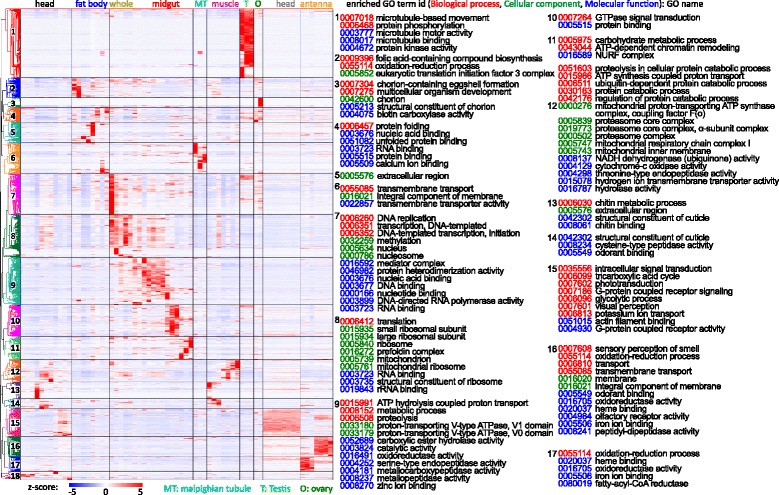
Library-specific expression of different genes in OGS2.0. Z-scores for highly expressed genes were calculated from FPKM values. Genes were clustered based on z-scores and divided to different groups manually based on the expression pattern. Significantly enriched GO terms (*p* < 0.05) for different clusters were labeled on the right, with GO numbers in red, green and blue represent Biological process, Cellular component and Molecular function, respectively

In theory, FPKM values are proportional to mRNA levels inside cells, especially for genes with high FPKM values. To acquire an overview of library-correlated gene expression, we prepared a heatmap of z-scores for genes with at least one FPKM value >100 and divided these 6108 genes into 18 clusters based on their expression patterns (Fig. 7). The 896 genes in cluster 1 are (more) highly expressed in the testis libraries, some highly expressed in all three and others either in T-P-D3 or in T-P-D15~18 and T-A-D1~3. Cluster 3 genes are (more) highly expressed in adult ovary (O-A-D1); cluster 7 in 3 h embryo; cluster 9, including many digestive enzyme genes, in larva midgut (data not shown); cluster 13 in pre-wandering head; clusters 16 and 17 in adult and larva antenna, respectively. Noncoding genes and MCOT specific genes show similar expression patterns (Additional file 2: Figure S1). The original z-scores for Fig. 7 and Additional file 2: Figure S1 can be found in (Additional file 9: Tables S7−S9).

To describe general features of the genes in different clusters, we performed a GO enrichment analysis (Fig. 7). As expected, the enriched GO terms were well correlated with the expression pattern of gene clusters. For example, the most significantly enriched terms of cluster 1 include microtubule binding, microtubule motor activity, and protein kinase activity under molecular function (Fig. 7). These terms suggest that meiosis and sperm generation in testis heavily use kinase cascades and microtubule-binding for chromosome movement. The upregulation of microtubule-related proteins was also observed in *D. melanogaster* [46]. Cluster 3 genes are more specifically expressed in adult ovaries, and the enriched GO terms, including chorion and eggshell formation, were tightly linked with female reproduction [47]. Similar clusters of genes more specifically expressed in testes and ovaries were also identified in *D. melanogaster* [38]. Cluster 7 genes are highly expressed in early embryo, and the enriched terms, including DNA replication, transcription and methylation, were tied to early embryonic development, same as observed in *D. melanogaster* [48].

### tRNA genes and codon usage

Different organisms have different codon preferences, which is associated with the age of genes and influenced by natural selection [49, 50]. Most codon frequency tables are calculated based on a limited number of protein sequences and might be inaccurate [51]. To complete this transcriptome study, we took advantage of the OGS2.0 and transcriptome data to examine a conceivable relationship between codon preferences and tRNA genes (Additional file 2: Table S10). For some codons, the predicted tRNA gene number is 0 (e.g. CTC), while its codon frequency in the genome and transcriptome is 15.2 and 17.7 per thousand, respectively. In other words, there is no direct correlation with tRNA gene numbers due to codon-anticodon wobbling. However, the tRNA gene number and codon frequency of different amino acids are highly correlated both in genome and transcriptome as shown in (Additional file 2: Figure S2). This suggests that the number of tRNAs might be regulated at the amino acid level instead of codon level. We also found the codon usages in genome and transcriptome generally agree. By calculating codon preferences in different libraries based on the RNA-seq data, we realized that codon usage is mainly influenced by highly expressed genes and there are no obvious global changes in codon preference.

## Discussion

We performed an in-depth analysis of the *M. sexta* RNA-seq libraries and described their qualities in terms of read composition. Data indicate that, in a few libraries, over 30% of the reads came from rRNA or mitochondrial genes (Fig. 2f) due to problems in sample preparation. We excluded these reads when calculating FPKM values because, otherwise, abundant rRNA and mitochondrial reads would greatly increase the total number of mapped reads and FPKM values of other genes would be lower than normal.

The Cufflinks4.0 genes and exons cover 51.7% and 17.1% of the genome, higher than those of OGS2.0 (41.6% and 9.2%). Preference for protein-coding genes during manual annotation, an important step of OGS2.0 [26], may have led to the difference. Some Cufflinks4.0 genes might be pseudo-genes predicted from pervasive transcribed parts of the genome [52, 53]. Cufflinks4.0 has 33,375 genes, a lot higher than 15,542 in OGS2.0. From the current data, 64% of the genome is transcribed, somewhat similar to 85% for human [52]. However, Cufflinks4.0 gene exons composed 17.1% of the genome of *M. sexta.* Compared with 4.1% of the human genome, the major difference between the exon region and transcribed region could be caused by deep sequencing of newly transcribed RNAs which may contain introns, failure in modeling some genes by Cufflinks, pervasive transcription of the genome [52, 53], and genomic DNA contamination.

Embryo, larva, pupa, and adult are the four distinct life stages of holometabolous insects and genes were differently expressed in various tissues/organs. The interlibrary comparisons showed large differences among 67 libraries (Fig. 5), as most parts of the plot are either white or grey. The red blocks off the diagonal line are mostly from libraries sharing tissues. For example, libraries 53–56 from female head with antenna are similar with libraries 64–66 from female antenna. We also note that library 5 (H-L5–12 h-S) is similar with libraries 43 (M-L5–12 h-S), but not libraries 42 (M-L5–12 h). The libraries 42 and 43 are from same tissue type, but sequenced differently, suggesting that other technical factors may also influence the interlibrary comparison.

The gene expression level based on OGS2.0, Cufflinks4.0 and MCOT1.0 each exhibited similar correlations with tissues and life stages (Fig. 7 and Additional file 2: Figure S1). Some genes, including the highly expressed ones (Fig. 6) are expressed at very high levels in one or a few closely-related libraries. Such sharp distinctions are seldom seen in *Drosophila* or mosquito studies, probably due to their small sizes and short larval and pupal stages. As clear separation of unrelated tissues or stages is a prerequisite for accurate identification of tissue/stage-specific genes, *M. sexta*, a large insect similar to many lepidopteran insects, is ideal for future tissue/stage-specific transcriptome analyses to serve as a better model for agricultural pest species.

Differentially expressed genes can provide useful leads for their functional elucidation. For example, the serine protease-102 is highly expressed in library of pupal ovary day 15 to 18 (Fig. 5), suggesting its function in female reproduction. Additionally, in-depth analyses of the RNA-seq data may reveal tissue/stage-specific alternative splicing and transcriptional co-regulation to guide functional investigations. To date, less than 15% of the OGS1.0 genes have been manually curated by a sizable community of experts [28]. While GO terms describe the general gene functions, we were only able to assign GO terms to 60% of the genes. By carefully examining individual gene groups along with homolog search and domain prediction, we expect an acceleration of gene function research in *M. sexta* and other insects.

## Conclusions

We comprehensively described and summarized the publicly available 67 RNA-seq libraries of *M. sexta*, explored the quality and mapping percentage of RNA-seq reads, explained the composition of RNA-seq reads and found rRNA and mitochondrial reads contributed remarkably to the mapped and unmapped groups. We named OGS2.0 genes, calculated and summarized FPKM values of gene expression in one table to facilitate their future functional studies. We compared similarities of various libraries and observed gene expression changed dramatically within days in libraries from the same tissues. Some most highly-expressed genes were unknown, with no homologs which have been studied. Most genes are library-correlated, including many highly-expressed genes which are highly library-specific. This is the first study which analyzed various transcriptome data from well-dissected tissues of an insect at different life stages. We hope it will greatly facilitate studies in *M. sexta* and other insects in the future.

## Additional files

Additional file 1: Table S1. Information about the 67 M. sexta cDNA libraries. Detailed information of the libraries, including SRA accession number, library type, total reads number, unmapped reads number, etc. (XLSX 19 kb)

Additional file 2: Tables **S3** and **S10, Figures S1** and **S2**. Statistics of the unmapped reads with BLASTN hits; Table S10. Codon usage in M. sexta; Additional file 2: Figure S1: Library-specific expression of MCOT1.0-specific and noncoding genes; Additional file 2: Figure S2. Relationship between codon usage in genome/transcriptome and tRNA gene numbers for different amino acids. (DOCX 789 kb)

Additional file 3: Transcript sequences of the coding genes in Cufflinks4.0. DNA sequences of the coding transcripts modeled by Cufflinks. (ZIP 20242 kb)

Additional file 4: Transcript sequences of the noncoding genes in Cufflinks4.0. DNA sequences of the noncoding transcripts modeled by Cufflinks. (ZIP 8122 kb)

Additional file 5: Table S2. Composition of unmapped reads in each of the 67 libraries. The table includes number of the reads matching different subject sequences in each of the 67 libraries. The first column is accession IDs of subject sequences and the second column is group of subject sequences. (XLSX 10110 kb)

Additional file 6: Table S4. Expression of genes in OGS2.0. FPKM values of genes in OGS2.0 calculated by RSEM. (XLSX 6733 kb)

Additional file 7: Table S5. Expression of genes in Cufflinks4.0. FPKM values of genes in Cufflinks4.0 calculated by RSEM. (XLSX 12927 kb)

Additional file 8: Table S6. Expression of genes in MCOT1.0. FPKM values of genes in MCOT1.0 calculated by RSEM. (XLSX 7331 kb)

Additional file 9: Tables **S7, S8** and **S9**. Z-score of high-expressed genes in OGS2.0 (Table S7), Cufflinks4.0 (Table S8), and MCOT1.0 (Table S9). Z-scores of genes with at least one FPKM value over 100 were calculated. The genes are in the same order as in Fig. 7, Additional file 2: **Figure S1A** and **S1B**, respectively. (XLSX 5418 kb)

## Abbreviations

A: Adult
An: Antenna
BPKM: Bases per kilobase per million mapped bases
CDS: Coding sequence
E: Embryo
F: Fat body
FDR: False discovery rate
FPKM: Fragments per kilobase per million mapped reads
G: Midgut
GO: Gene ontology
H: Head
L: Larval
M: Muscle
MCOT: A gene set generated by integrating the best models from the programs MAKER2, Cufflinks, Oases and Trinity
MT: Malpighian tubule
NGS: Next-generation sequencing
O: Ovary
OGS: Official gene set
P: Pupal
T: Testis
W: Wandering
W: Whole body
