# Supplementary material for: An analysis of 67 RNA-seq datasets from various tissues at different stages of a model insect, Manduca sexta
Source: BMC Genomics. 2017 Oct 17;18:796. doi: 10.1186/s12864-017-4147-y (PMC5645894; doi:10.1186/s12864-017-4147-y)
Supplement: Supplementary file 2 — S3 and S10, Figures S1 and S2. Statistics of the unmapped reads with BLASTN hits; Table S10. Codon usage in M. sexta; Additional file 2: Figure S1: Library-specific expression of MCOT1.0-specific and noncoding genes; Additional file 2: Figure S2. Relationship between codon usage in genome/transcriptome and tRNA gene numbers for different amino acids. (DOCX 789 kb) [file 12864_2017_4147_MOESM2_ESM.docx]

**Table S1.** Detailed information on the cDNA libraries (*A portion of the table is here to help the review*.)

| Library # | Library group | Library name | Development stage | NCBI SRA accession # | # of reads | Length | Total bases | Reads type | Overall trimming survived reads | TopHat mapped reads | TopHat mapped bases | Transcribed genome length | Transcribed genome (%) | Mapped reads STAR | Unmapped reads STAR | Unmapped w. BLASTN match |
| --- | --- | --- | --- | --- | --- | --- | --- | --- | --- | --- | --- | --- | --- | --- | --- | --- |
| 1 | P | H-L2-D1 | Head, 2^nd^ instar, day 1 | SRX702730 | 3.29E+7 | 100 | 3.29E+9 | paired | 3.06E+7 | 2.63E+7 | 2.49E+9 | 5.79E+7 | 13.81 | 2.87E+7 | 1.95E+6 | 1.50E+6 |
| 2 | P | H-L3-D1 | Head, 3^rd^ instar, day 1 | SRX702731 | 4.02E+7 | 100 | 4.02E+9 | paired | 3.68E+7 | 3.16E+7 | 2.99E+9 | 6.43E+7 | 15.32 | 3.40E+7 | 2.83E+6 | 2.31E+6 |
| … | … | … | … | … | … | … | … | … | … | … | … | … | … | … | … | … |

**Table S3.** Statistics of the unmapped reads with BLASTN hits*

| Category | Read number | Percentage (%) |
| --- | --- | --- |
| rRNA | 76,843,832 | 80.23 |
| mitochondrion | 1,714,581 | 1.79 |
| phage | 6,582,176 | 6.87 |
| *M. sexta* | 2,675,232 | 2.79 |
| *E. coli* | 1,605,683 | 1.68 |
| *Oryza* | 1,080,110 | 1.12 |
| other | 5,271,964 | 5.50 |
| Grand total | 95,773,578 | 100 |

* Total number of the unmapped reads in all the 67 cDNA libraries.

**Table S10.** Codon usage in *M. sexta*

|  | T | | | | | C | | | | | A | | | | | G | | | | |  |
| --- | --- | --- | --- | --- | --- | --- | --- | --- | --- | --- | --- | --- | --- | --- | --- | --- | --- | --- | --- | --- | --- |
|  | codon | AA | freq. g ^a^ | freq. t ^b^ | tRNA # ^c^ | codon | AA | freq. g | freq. t | tRNA # | codon | AA | freq. g | freq. t | tRNA # | codon | AA | freq. g | freq. t | tRNA # |  |
| T | TTT | F | 13.7 | 11.0 | 1 | TCT | S | 12.6 | 12.3 | 19 | TAT | Y | 12.7 | 10.4 | 1 | TGT | C | 9.1 | 6.1 | 0 | T |
|  | TTC | F | 20.8 | 24.8 | 26 | TCC | S | 11.3 | 13.4 | 0 | TAC | Y | 19.0 | 22.6 | 28 | TGC | C | 11.9 | 10.5 | 21 | C |
|  | TTA | L | 14.5 | 10.7 | 14 | TCA | S | 12.8 | 9.8 | 9 | TAA | - ^d^ | 0.9 | 2.0 | 0 | TGA | - ^d^ | 0.6 | 1.2 | 1 ^e^ | A |
|  | TTG | L | 16.7 | 15.6 | 16 | TCG | S | 13.9 | 9.2 | 15 | TAG | - ^d^ | 0.4 | 0.6 | 3 ^f^ | TGG | W | 11.0 | 9.2 | 8 | G |
| C | CTT | L | 10.3 | 11.7 | 15 | CCT | P | 13.3 | 14.1 | 28 | CAT | H | 10.7 | 9.0 | 0 | CGT | R | 6.6 | 9.3 | 24 | T |
|  | CTC | L | 15.2 | 17.7 | 0 | CCC | P | 11.9 | 16.1 | 0 | CAC | H | 15.0 | 14.1 | 23 | CGC | R | 13.9 | 13.1 | 0 | C |
|  | CTA | L | 9.2 | 7.0 | 6 | CCA | P | 15.0 | 13.4 | 17 | CAA | Q | 19.1 | 17.1 | 23 | CGA | R | 7.1 | 4.9 | 14 | A |
|  | CTG | L | 22.5 | 20.1 | 18 | CCG | P | 16.5 | 10.4 | 11 | CAG | Q | 19.6 | 20.2 | 20 | CGG | R | 7.3 | 4.7 | 0 | G |
| A | ATT | I | 15.5 | 15.4 | 27 | ACT | T | 14.9 | 13.2 | 23 | AAT | N | 22.4 | 19.0 | 1 | AGT | S | 12.2 | 9.6 | 1 | T |
|  | ATC | I | 17.1 | 24.0 | 0 | ACC | T | 13.1 | 15.4 | 4 | AAC | N | 24.3 | 25.8 | 41 | AGC | S | 13.7 | 11.9 | 16 | C |
|  | ATA | I | 19.3 | 12.0 | 11 | ACA | T | 16.3 | 14.5 | 28 | AAA | K | 34.1 | 33.6 | 22 | AGA | R | 13.0 | 10.9 | 9 | A |
|  | ATG | M | 22.6 | 22.3 | 44 | ACG | T | 14.0 | 9.3 | 11 | AAG | K | 27.8 | 40.5 | 26 | AGG | R | 9.8 | 10.9 | 13 | G |
| G | GTT | V | 13.3 | 14.9 | 24 | GCT | A | 17.5 | 23.3 | 33 | GAT | D | 25.2 | 23.8 | 6 | GGT | G | 13.6 | 19.7 | 1 | T |
|  | GTC | V | 14.1 | 17.8 | 0 | GCC | A | 17.8 | 24.8 | 0 | GAC | D | 29.8 | 32.5 | 54 | GGC | G | 21.0 | 23.3 | 32 | C |
|  | GTA | V | 12.1 | 12.8 | 12 | GCA | A | 14.3 | 14.3 | 23 | GAA | E | 34.2 | 34.1 | 33 | GGA | G | 14.7 | 17.1 | 18 | A |
|  | GTG | V | 25.3 | 24.7 | 24 | GCG | A | 23.0 | 17.5 | 22 | GAG | E | 31.0 | 32.4 | 28 | GGG | G | 7.9 | 7.0 | 3 | G |

^a^: freq. g, frequency per thousand based on CDS of gene; ^b^: freq. t: frequency based on ORF in transcript; ^c^: number of tRNA genes with corresponding anticodon; ^d^: “-” for stop codon; ^e^: seleno-Cys tRNA gene; f, suppressor tRNA gene.

**Fig. S1.** **Library-specific expression of MCOT1.0-specific (A) and noncoding (B) genes**. Z-scores for highly expressed genes were calculated from FPKM values. Genes were clustered based on z-scores and divided to different groups manually based on the expression pattern.

**Fig. S2.** **Relationship between codon usage in genome (green)/transcriptome (red) and tRNA gene number for different amino acids**. Amino acids in single letter were labeled in the figure. Linear regression was performed for genome/transcriptome, with R^2^ labeled in the figure.
